# Sintomas Cardiopulmonares Pós-COVID-19: Preditores e Características de Imagem de Pacientes após a Alta Hospitalar

**DOI:** 10.36660/abc.20220642

**Published:** 2023-05-18

**Authors:** Kalil-Filho Roberto, Roberta Saretta, André Franci, Luciano M. Baracioli, Filomena R. B. G. Galas, Juliana S. Gil, Amanda Ferino, Camilla Giacovone, Isabella Oliveira, Johnatan Souza, Vanessa Batista, Augusto Scalabrini, Livia do Valle Costa, Amanda Danieleto Ruiz, Carla B. Ledo, Teresa Cristina D. C. Nascimento, Luciano F. Drager

**Affiliations:** 1 Hospital Sírio Libanês São Paulo SP Brasil Hospital Sírio Libanês, São Paulo, SP – Brasil; 2 Hospital das Clínicas Faculdade de Medicina Universidade de São Paulo São Paulo SP Brasil Instituto do Coração (InCor), Hospital das Clínicas da Faculdade de Medicina da Universidade de São Paulo, São Paulo, SP – Brasil

**Keywords:** COVID-19, Sinais e Sintomas, Sexo, Depressão

## Abstract

**Fundamento:**

A maioria da evidência sobre o impacto da síndrome COVID pós-aguda (PACS, do inglês, *post-acute* COVID-19 syndrome) descreve sintomas individuais sem correlacioná-los com exames de imagens.

**Objetivos:**

Avaliar sintomas cardiopulmonares, seus preditores e imagens relacionadas em pacientes com COVID-19 após alta hospitalar.

**Métodos:**

Pacientes consecutivos, que sobreviveram à COVID-19, foram contatados 90 dias após a alta hospitalar. A equipe de desfechos clínicos (cega quanto aos dados durante a internação) elaborou um questionário estruturado avaliando sintomas e estado clínico. Uma análise multivariada foi realizada abordando a evolução da COVID-19, comorbidades, ansiedade, depressão, e estresse pós-traumático durante a internação, e reabilitação cardíaca após a alta. O nível de significância usado nas análises foi de 5%.

**Resultados:**

Foram incluídos 480 pacientes (idade 59±14 anos, 67,5% do sexo masculino) que receberam alta hospitalar por COVID-19; 22,3% necessitaram de ventilação mecânica. A prevalência de pacientes com sintomas cardiopulmonares relacionados à PACS (dispneia, cansaço/fadiga, tosse e desconforto no peito) foi de 16,3%. Vários parâmetros de tomografia computadorizada do tórax e de ecocardiograma foram similares entre os pacientes com e sem sintomas cardiopulmonares. A análise multivariada mostrou que sintomas cardiopulmonares foram relacionados de maneira independente com sexo feminino (OR 3,023; IC95% 1,319-6,929), trombose venosa profunda durante a internação (OR 13,689; IC95% 1,069-175,304), nível elevado de troponina (OR 1,355; IC95% 1,048-1,751) e de proteína C reativa durante a internação (OR 1,060; IC95% 1,023-1,097) e depressão (OR 6,110; IC95% 2,254-16,558).

**Conclusão:**

Os sintomas cardiopulmonares relacionados à PACS 90 dias após a alta hospitalar são comuns e multifatoriais. Além dos marcadores trombóticos, inflamatórios e de lesão miocárdica durante a internação, sexo feminino e depressão foram associados independentemente com sintomas cardiopulmonares relacionados à PACS. Esses resultados destacaram a necessidade de uma abordagem multifacetada direcionada a pacientes susceptíveis.

## Introdução

A COVID-19 (do inglês, *coronavirus disease* 2019), uma doença sistêmica causada pelo coronavírus da síndrome respiratória aguda grave 2 (SARS-CoV-2), tornou-se um dos grandes desafios à população global.^1^ Em 30 de junho de 2022, foram registrados 548 101 683 casos, com 6 337 024 mortes confirmadas, que representa uma mortalidade global de 1,16%.^2^ Pelo fato de a maioria dos pacientes terem sobrevivido à COVID-19, há um interesse crescente acerca dos potenciais efeitos subagudos e de longo prazo da COVID-19.^3^ A maioria dos estudos tem abordado os sintomas persistentes nos pacientes após a fase aguda da doença [síndrome COVID pós-aguda (PACS, do inglês, *post-acute COVID-19 syndrome* )],^3 - 5^ enquanto outros relataram o impacto da COVID-19 sobre imagem torácica ou cardíaca, sem fazer referência aos sintomas.^6 - 8^

Apesar dessa evidência prévia, existem várias lacunas potenciais nesta área de pesquisa que merecem atenção especial.^9^ Primeiramente, é crucial explorar a frequência e os determinantes da PACS por meio de uma abordagem abrangente que inclua não somente a evolução da COVID-19 como também outros fatores como idade, e condições pré-existentes como doenças cardíacas, pulmonares e mentais após a alta hospitalar. Ainda, a maioria dos estudos sobre o impacto da PACS não enfatizou sintomas cardiopulmonares nem os correlacionou com achados de exames de imagem.

O principal objetivo do estudo foi avaliar a magnitude e os preditores independentes dos sintomas cardiopulmonares relacionados à PACS 90 dias após a alta hospitalar em pacientes consecutivos. Além disso, imagens cardiopulmonares disponíveis durante e após a internação hospitalar foram correlacionadas com sintomas cardiopulmonares. A principal hipótese testada baseou-se na premissa de que os sintomas cardiopulmonares podem ter múltiplas causas, não limitadas à gravidade da COVID-2 durante a internação, e sim potencialmente correlacionadas ao sexo e a distúrbios mentais.

## Métodos

Os dados que corroboram os achados deste estudo estão disponíveis com o autor de correspondência mediante devida solicitação.

O estudo foi analisado pelo Comitê de Ética da instituição e dispensado de sua provação (Instituto de Ensino e Pesquisa, IEP, Hospital Sírio Libanês). Todos os dados foram analisados em um banco de dados seguro, anônimo e separado do servidor principal. Não houve envolvimento dos pacientes ou do público no delineamento, desenvolvimento, relato ou disseminação da pesquisa. Pacientes hospitalizados consecutivos foram recrutados no Hospital Sírio Libanês, de março de 2020 a abril de 2021. Todos os pacientes tiveram um diagnóstico confirmado de COVID-19 pela presença de sintomas relacionados e um resultado positivo de PCR (reação em cadeia da polimerase) para SARS-CoV-2 tanto para amostra coletada por *swab* nasal como por *swab* de faringe.

Todos os dados foram revisados pela equipe do estudo para assegurar a acurácia. O registro utilizou-se de um formulário de relato de caso *online* , disponível na plataforma RedCap^TM^ (Nashville, TN, US). REDCap é uma aplicação web para a construção e gerenciamento de banco de dados e inquéritos online (https://www.project-redcap.org/).

### Variáveis e desfechos clínicos coletados no hospital

Os dados dos participantes foram coletados até a alta hospitalar. Características demográficas, história e apresentação clínica, resultados laboratoriais (incluindo valores mais altos de troponina I, e níveis de dímeros D, proteína C reativa, e creatinina sérica), dias de internação, necessidade de ventilação mecânica, oxigenação por membrana extracorpórea (ECMO), choque séptico pulmonar, embolismo pulmonar, trombose venosa profunda, sangramento maior, necessidade de diálise, e medicamentos usados durante a internação (incluindo antibióticos, anticoagulante, plasma convalescente, corticoides e cloroquina/hidroxicloroquina) foram coletados para análise. Ainda, dados de tomografia computadorizada (TC) do tórax e de ecocardiograma transtorácica foram documentados em um subgrupo de pacientes com exames disponíveis. Para pacientes com mais de uma TC do tórax durante a internação, o exame indicando o maior envolvimento pulmonar foi usado na análise. Para quantificar a extensão das anormalidades pulmonares (lesões totais, consolidação, reticulação, alterações fibróticas), um escore de TC semiquantitativo foi aplicado, com base na área comprometida em cada um dos lóbulos pulmonares, atribuindo-se a pontuação – 0, sem comprometimento, 1, menos que 5% de comprometimento; 2, 5-25% de comprometimento; 3, 26-49% de comprometimento; 4, 50-75% de comprometimento; e 5, mais de 75% de comprometimento.^10^

### Variáveis coletadas após a alta hospitalar

Os desfechos clínicos após a alta hospitalar foram avaliados por meio de um questionário padronizado 90 dias após a alta. O questionário foi aplicado por uma equipe que não tinha acesso às variáveis hospitalares. A presença de sintomas cardiovasculares (autorrelatados) como dispneia, cansaço, fadiga, tosse, e desconforto torácico foram considerados como desfechos cardiopulmonares do PACS. Como cansaço/fadiga pode representar um sintoma cardiopulmonar inespecífico, realizou-se uma sub-análise considerando somente dispneia, tosse e desconforto torácico. Ainda, dados descritivos de outros sintomas como anosmia, disgeusia, cefaleia, artralgia, mialgia e diarreia foram incluídos nos resultados. Todos os pacientes foram avaliados quanto à participação em programa de reabilitação cardiovascular (sim/não), estado funcional, reinternações hospitalares (incluindo por sintomas relacionados à COVID-19), eventos cardiopulmonares, necessidade de oxigênio e de diálise. Ainda, o questionário *European Quality of Life Five Dimension* (EQ-5D) foi aplicado para avaliar qualidade de vida. Essa ferramenta gratuita (https://euroqol.org/) abrange cinco domínios da saúde (mobilidade, cuidado pessoal, atividades habituais, dor/desconforto, ansiedade/depressão), permitindo três níveis de resposta, e uma escala analógica visual, usada para autoavaliação da saúde do paciente. A pontuação do EQ-5D varia entre 0 e 1, em que 1 representa a melhor qualidade de vida. A escala analógica visual varia de 0 a 100, sendo 100 o melhor estado de saúde imaginável, e 0 o pior estado de saúde imaginável.^11^

### Transtorno do estresse pós-traumático

O transtorno do estresse pós-traumático relacionado à internação por COVID-19 foi objetivamente avaliada pela pergunta: “Você tem pensamentos, memórias ou imagens repetitivas e perturbadoras sobre a experiência da doença/hospitalização recente?” “Você apresenta sintomas físicos (batimentos acelerados, sudorese, dificuldade em respirar) ao se lembrar da experiência de internação/doença?”.

### Ansiedade ( *Generalized Anxiety Disorder 2-item, GAD-2* )

O GAD-2 é uma escala simplificada validada usada para definir um transtorno de ansiedade generalizada.^12^ Cada paciente foi convidado a responder as seguintes perguntas: “Nos últimos dois anos, com que frequência você se sentiu incomodado com os seguintes problemas? 1) Sentindo-se nervoso, ansioso ou além dos seus limites; 2) Não se sentir capaz de interromper ou controlar suas preocupações”. Para cada pergunta, uma das seguintes respostas foi escolhida: Nunca (0 ponto); vários dias (1 ponto); mais da metade dos dias (2 pontos); quase diariamente (3 pontos). Com um ponto de corte de 3 pontos (ou mais), o GD-2 tem sensibilidade de 86% e especificidade de 83% para diagnosticar transtorno de ansiedade generalizada.

### Depressão ( *Patient Health Questionnaire-2* , PHQ-2)

O PHQ-2 é um instrumento usado para investigar a frequência de humor depressivo e anedonia ao longo dos dois últimos anos. Cada paciente foi solicitado a responder as seguintes perguntas: ao longo das duas semanas, com que frequência você se sentiu incomodado com os seguintes problemas? 1) Pouco interesse ou prazer em realizar atividades; 2) Sentindo-se para baixo, deprimido ou desesperançoso. Para cada pergunta, uma das seguintes respostas foi escolhida: nunca (0 ponto); vários dias (1 ponto); mais da metade dos dias (2 pontos); quase diariamente (3 pontos). A pontuação varia entre 0 e 6; um escore igual ou maior que três indica provável presença de transtorno depressivo maior.^13^

### Análise estatística

As variáveis contínuas foram descritas em média e desvio padrão ou mediana e intervalo interquartil, de acordo com a normalidade dos dados (teste de Shapiro-Wilk). As variáveis categóricas foram descritas em frequência relativa e absoluta. Na análise univariada, comparações entre pacientes com e sem sintomas cardiopulmonares foram feitas pelo teste do qui-quadrado para variáveis categóricas e teste t não pareado/Mann Whitney para variáveis contínuas (de acordo com distribuição paramétrica e não paramétrica, respectivamente). Análise de regressão logística multivariada foi realizada para detectar preditores independentes de sintomas cardiopulmonares 90 dias após a alta hospitalar, considerando fatores basais, achados laboratoriais e variáveis pós-alta hospitalar. As variáveis com um valor de p<0,2 nas análises univariadas (análise de uma variável por vez) foram incluídas no modelo final.^14^ Ainda, consideramos as variáveis com relevância biológica para os desfechos principais, incluindo idade, índice de massa corporal, tabagismo, doença pulmonar obstrutiva crônica/asma, e doença cardiovascular prévia. Para depressão e ansiedades, os dados dos questionários validados (e não de diagnósticos autorrelatados) foram usados para as análises. O nível de significância usado foi de 5%. Toda as análises foram realizadas usando o programa IBM-SPSS (Statistical Package for the Social Sciences, SPSS, Inc, Chicago, IL) versão 24.

## Resultados

**Figura 1:**
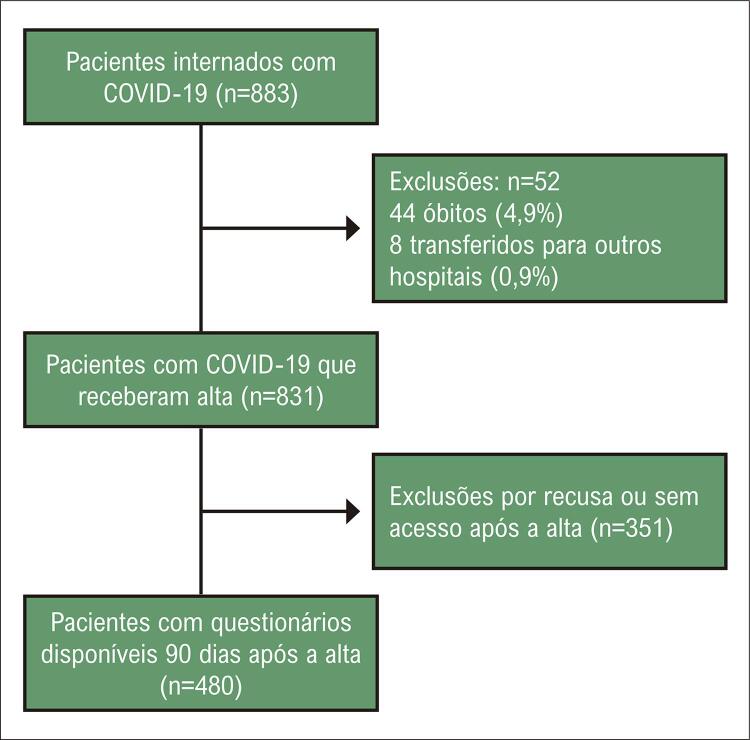
– Fluxograma do estudo.

Um total de 883 pacientes foram inicialmente rastreados. Após excluir os pacientes que foram a óbito durante a internação e aqueles transferidos a outros hospitais, 831 tiveram alta hospitalar ( Figura 1 ). Características dos pacientes incluídos (n=480) e excluídos (n=351) estão descritas na Tabela S1 (material suplementar *online* ). Embora vários parâmetros não tenham sido diferentes entre os dois grupos, os pacientes incluídos na análise eram mais velhos, apresentaram maior prevalência de doenças cardiovasculares, menor frequência de depressão autorrelatada, maior permanência hospitalar, maior necessidade de diálise, e os níveis mais altos de dímero-D e proteína C reativa durante internação em comparação aos pacientes sem dados após hospitalização. A Tabela 1 apresenta as principais características dos pacientes incluídos na análise; os pacientes eram predominantemente homens, brancos, com sobrepeso, e várias comorbidades como hipertensão, diabetes e dislipidemia. Quase um quarto dos pacientes estavam na Unidade de Terapia Intensiva (UTI). Como esperado para esses pacientes, eles apresentaram níveis elevados de marcadores trombóticos e inflamatórios.

**Tabela 1:** – Características dos pacientes durante internação hospitalar por COVID-19 e após a alta

Características	N=480

Características demográficas, antropométricas e comorbidades	
Idade (anos)	59 ± 14
Homens, n (%)	324 (67,5)
Raça branca (autorrelatada), n (%)*	397 (98)
Índice de massa corporal (kg/m^2^)	27,9 (25,5 – 30,8)
Alcoolismo, n (%)	16 (3,3)
Tabagismo atual, n (%)	26 (5,4)
Hipertensão, n (%)	202 (42,1)
Diabetes, n (%)	108 (22,5)
Dislipidemia, n (%)	130 (27,1)
Doença cardiovascular prévia, n (%)	142 (29,6)
Doença cerebrovascular prévia, n (%)	11 (2,3)
DPOC / asma, n (%)	41 (8,5)
Doença renal crônica, n (%)	13 (2,7)
Diagnóstico prévio de ansiedade, n (%)	13 (2,7)
Diagnóstico prévio de depressão, n (%)	13 (2,7)

**Dados durante a internação**	
Tempo de internação, dias	10 (7 - 16)
Unidade de terapia intensiva, n (%)	107 (22,3)
Ventilação mecânica, n (%)	62 (12,9)
Oxigenação por membrana extracorpórea n (%)	7 (1,5)
Embolia pulmonar, n (%)	7 (1,5)
Trombose venosa profunda, n (%)	6 (1,3)
Diálise, n (%)	4 (0,8)
Sangramento maior, n (%)	11 (2,3)
Polineuropatia do paciente crítico, n (%)	19 (4,0)
Nível mais alto de troponina I (ng/mL)	0,15 (0,15 – 0,15)
Nível mais alto de dímero-D (ng/mL)	716 (410 - 1290)
Nível mais alto de proteína C-reativa (mg/L)	4,63 (1,52 – 11,54)
Nível mais alto de creatinina (mg/dL)	1,03 (0,86 – 1,20)
Antibióticos, n (%)	480 (100)
Plasma convalescente, n (%)	14 (2,9)
Anticoagulantes profiláticos ou terapêuticos, n (%)	455 (94,8)
Corticoides, n (%)	362 (75,4)
Cloroquina / hidroxicloroquina, n (%)	99 (20,6)

* n=405; DPOC: doença pulmonar obstrutiva crônica.

**Figura 2:**
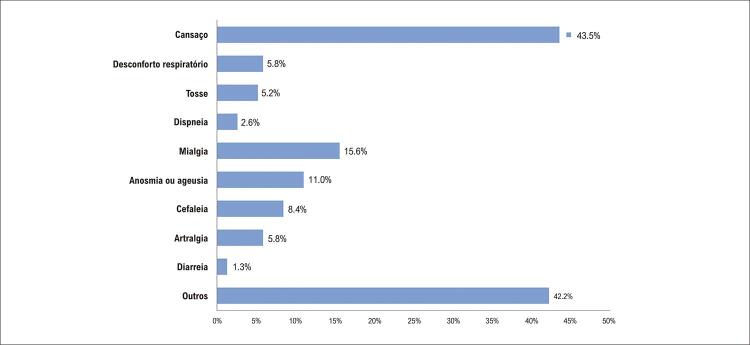
– Frequência de sintomas relatados pelos pacientes 90 dias após a alta hospitalar por COVID-19.

A prevalência dos pacientes com quaisquer desses sintomas foi 32,1% (n=154) e a de pacientes com sintomas cardiopulmonares relacionados à PACS 90 dias após a alta hospitalar foi 16,3% (n=78). O sintoma mais comum foi cansaço, seguido de desconforto respiratório, tosse e dispneia. A distribuição detalhada dos sintomas está descrita na Figura 2 . Os pacientes com sintomas cardiopulmonares aos 90 dias apresentaram frequência mais alta de depressão, internação hospitalar mais longa, maior necessidade de ventilação mecânica e de terapia intensiva, maior taxa de polineuropatia do paciente crítico, e maiores níveis de proteína C reativa durante internação em comparação a pacientes sem esses sintomas ( Tabela 2 ). A Tabela 3 apresenta dados sobre os questionários estruturados, reabilitação cardiovascular pós-alta hospitalar e reinternação por COVID-19. Os pacientes com sintomas cardiopulmonares apresentaram pior qualidade de vida, maior taxa de ansiedade (GAD-2), depressão (PHQ-2), e transtorno de estresse pós-traumático em comparação a pacientes sem esses sintomas. Não observamos diferença na taxa de reinternação hospitalar por COVID-19.

**Tabela 2:** – Características dos pacientes com e sem sintomas cardiopulmonares (cansaço, dispneia, desconforto respiratório, tosse) 90 dias após internação por COVID-19

Características	Sem sintomas cardiopulmonares (n=402)	Com sintomas cardiopulmonares (n=78)	p
Características demográficas, antropométricas e comorbidades			
Idade (anos)	58,6 ± 14,7	60,0 ± 11,4	0,311
Homens, n (%)	278 (69,2)	46 (59,0)	0,079
Raça branca (autorrelatada), n (%)*	329 (98,2%)	68 (97,1)	0,363
Índice de massa corporal (kg/m^2^)	27,8 (25,6 – 30,8)	28,1 (24,3 – 30,4)	0,477
Alcoolismo, n (%)	12 (3,0)	4 (5,1)	0,309
Tabagismo atual, n (%)	23 (5,7)	3 (3,8)	0,784
Hipertensão, n (%)	172 (42,8)	30 (38,5)	0,479
Diabetes, n (%)	87 (21,6)	21 (26,9)	0,307
Dislipidemia, n (%)	108 (26,9)	22 (28,2)	0,808
Doença cardiovascular prévia, n (%)	118 (29,4)	24 (30,8)	0,802
Doença cerebrovascular prévia, n (%)	10 (2,5)	1 (1,3)	>0,999
DPOC / asma, n (%)	32 (8,0)	9 (11,5)	0,301
Doença renal crônica, n (%)	11 (2,7)	2 (2,6)	>0,999
Diagnóstico prévio de ansiedade, n (%)	9 (2,2)	4 (5,1)	0,241
Diagnóstico prévio de depressão, n (%)	8 (2,0)	5 (6,4)	0,044

**Dados durante a internação**			
Tempo de internação, dias	10 (7 – 15)	12 (8 – 22)	0,003
Unidade de terapia intensiva, n (%)	83 (20,6)	24 (30,8)	0,049
Ventilação mecânica, n (%)	43 (10,7)	19 (24,4)	0,001
Oxigenação por membrana extracorpórea n (%)	5 (1,2)	2 (2,6)	0,318
Embolia pulmonar, n (%)	5 (1,2)	2 (2,6)	0,318
Trombose venosa profunda, n (%)	3 (0,7)	3 (3,8)	0,086
Diálise, n (%)	3 (0,7)	1 (1,3)	0,509
Sangramento maior, n (%)	7 (1,7)	4 (5,1)	0,086
Polineuropatia do paciente crítico, n (%)	12 (3,0)	7 (9,0)	0,022
Nível mais alto de troponina I (ng/mL)	0,15 (0,15 – 0,15)	0,15 (0,15 – 0,16)	0,149
Nível mais alto de dímero-D (ng/mL)	705 (391 - 1289)	754 (458 - 1300)	0,122
Nível mais alto de proteína C-reativa (mg/L)	4,23 (1,39 – 10,16)	8,31 (2,52 – 18,98)	0,006
Nível mais alto de creatinina (mg/dL)	1,04 (0,87 – 1,20)	0,99 (0,83 – 1,19)	0,271
Antibióticos, n (%)	402 (100)	78 (100)	1,00
Plasma convalescente, n (%)	10 (2,5)	4 (5,1)	0,259
Anticoagulantes profiláticos ou terapêuticos, n (%)	381 (94,8)	74 (94,9)	>0,999
Corticoides, n (%)	299 (74,4)	63 (80,8)	0,230
Cloroquina / hidroxicloroquina, n (%)	80 (19,9)	19 (24,4)	0,373

* n=405; DPOC: doença pulmonar obstrutiva crônica.

**Tabela 3:** – Dados de qualidade de vida, ansiedade, depressão, estresse pós-traumático, e reabilitação cardiovascular 90 dias após alta hospitalar, e reinternação por COVID-19 nos pacientes com e sem sintomas cardiopulmonares (cansaço, dispneia, desconforto respiratório, tosse)

Características	Sem sintomas cardiopulmonares (n=402)	Com sintomas cardiopulmonares (n=78)	p
Qualidade de vida (EuroQol 5) *	1 (0,85 – 1,00)	0,85 (0,73 – 1,00	<0,001
Ansiedade (GAD-2), n (%) *	18 (5,4)	11 (16,9)	0,003
Depressão (PHQ-2), n (%) *	18 (5,3)	15 (23,1)	<0,001
Transtorno do estresse pós-traumático, n (%)*	4 (1,2)	5 (7,7)	0,013
Readmissão por COVID-19, n (%)	2 (0,5)	1 (1,3)	0,413

*Alguns pacientes não relataram essas condições.

**Figura 3:**
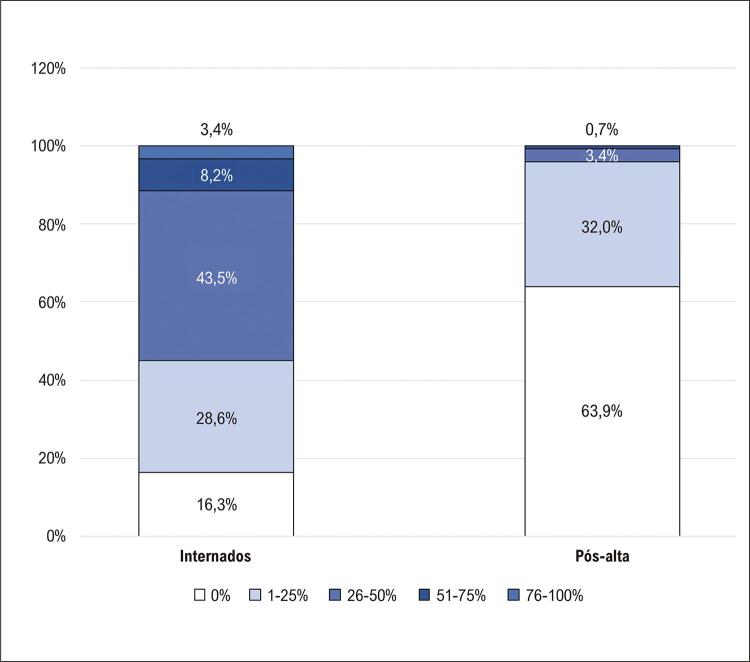
– Porcentagem de envolvimento pulmonar em pacientes com COVID-19 durante a internação hospitalar e após a alta.

Em um subgrupo de pacientes com exames de TC disponíveis, a maioria dos pacientes não apresentou anormalidades no pulmão após a alta ( Figura 3 ). Um achado interessante foi que vários parâmetros da TC do tórax e ecocardiograma transtorácico não foram diferentes entre os grupos com e sem sintomas cardiopulmonares (Tabela S2, material suplementar).

A análise multivariada mostrou que os sintomas cardiopulmonares relacionados à PACS foram associados independentemente com sexo feminino, trombose venosa profunda durante a internação, níveis mais altos de troponina I e proteína C reativa, e depressão (PHQ-2) ( Tabela 4 ). As outras variáveis incluídas no modelo não foram significativas (Tabela S3, material suplementar).

**Tabela 4:** – Análise multivariada avaliando as variáveis independentes associadas a sintomas cardiopulmonares (cansaço, dispneia, desconforto respiratório, tosse) 90 dias após alta hospitalar por COVID-19

	Coeficiente	OR	Intervalo de confiança de 95%		p
Idade (por ano)	0,001	1,001	0,999	1,003	0,355
Índice de massa corporal (para cada 1kg/m^2^)	0,005	1,005	0,999	1,010	0,081
Sexo (feminino)	1,106	3,023	1,319	6,929	0,009
Tabagismo (sim)	0,615	1,850	0,358	9,568	0,463
DPOC / asma (sim)	0,402	1,495	0,465	4,811	0,500
Doença cardiovascular prévia (sim)	0,478	1,612	0,743	3,499	0,227
Trombose venosa profunda (sim)	2,617	13,689	1,069	175,304	0,044
Nível mais alto de troponina I (ng/mL)	0,304	1,355	1,048	1,751	0,020
Nível mais alto de proteína C reativa (ng/mL)	0,058	1,060	1,023	1,097	0,001
Depressão (PHQ-2) (sim)	1,810	6,110	2,254	16,558	<0,001
Constante	-5,444				<0,001

DPOC: doença pulmonar obstrutiva crônica.

Uma subanálise excluindo cansaço como um sintoma cardiopulmonar revelou que somente 4,4% dos pacientes apresentaram dispneia, tosse, ou desconforto torácico aos 90 dias (Tabelas S4 e S5, material suplementar). A análise multivariada mostrou que depressão, alcoolismo, dias de hospitalização e uso de tocilizumabe mostraram relação independente com um ou mais desses sintomas (Tabela S6, material suplementar).

## Discussão

Este estudo avaliando os sintomas cardiopulmonares da PACS 90 dias após a alta hospitalar de pacientes consecutivos apresenta os seguintes resultados: 1) Os sintomas cardiopulmonares relacionados à PACS estão presentes em 16% dos pacientes; contudo, quando cansaço e fadiga não foram considerados na análise, menos de 5% dos pacientes relataram dispneia, tosse ou desconforto torácico; 2) não foram observadas alterações significativas na função do ventrículo esquerdo, e a maioria dos pacientes apresentaram uma tomografia normal no seguimento. Vários parâmetros de imagem não foram diferentes entre pacientes com e sem sintomas cardiopulmonares; 3) a causa dos sintomas cardiopulmonares da PACS é multifatorial. Além dos marcadores trombóticos, inflamatórios e de lesão miocárdica relacionados à gravidade da COVID-19 durante a internação, sexo feminino e presença de depressão são explicações possíveis para os sintomas ( Figura Central ). Nosso estudo destaca a necessidade de se identificarem fenótipos susceptíveis associados aos sintomas cardiopulmonares da PACS para se definirem estratégias preventivas por meio de uma abordagem multifacetada.

**Figura Central:**
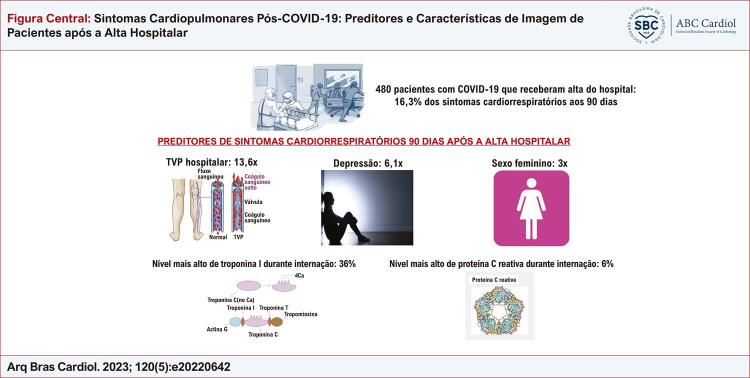
: Sintomas Cardiopulmonares Pós-COVID-19: Preditores e Características de Imagem de Pacientes após a Alta Hospitalar

A COVID-19 é frequentemente associada com inúmeros estímulos inflamatórios e pró-trombóticos, contribuindo para um pior prognóstico durante a fase hospitalar.^15 , 16^ Entretanto, o impacto desses estímulos sobre a recuperação pós-COVID-19 ainda não está claro. Em nosso estudo, a trombose venosa profunda e os níveis elevados de troponina I e proteína C reativa foram associados de maneira independente com os sintomas cardiopulmonares. Estudos anteriores sugeriram que o risco de tromboembolismo associado à internação parece estender-se até os primeiros 90 dias após a alta hospitalar, desde o momento da admissão.^17 - 19^ Tais resultados sugerem potenciais efeitos residuais que possam contribuir para os sintomas cardiopulmonares no acompanhamento em curto prazo. Contudo, a explicação da relação entre esses marcadores hospitalares e os sintomas cardiopulmonares não está bem esclarecida. Em nosso estudo, vários achados de imagem (incluindo o grau de comprometimento do pulmão, congestão pulmonar, derrame pleural e pericárdico, fração de ejeção do ventrículo esquerdo, alterações na contratilidade regional do ventrículo esquerdo, pressão arterial pulmonar, e presença de hipertensão pulmonar) não foram diferentes entre pacientes com e sem sintomas cardiopulmonares (Tabela S2, material suplementar). Nesse cenário, alterações estruturais promovidas por trombose venosa profunda (todas tratadas adequadamente durante a internação) podem não explicar totalmente os sintomas relacionados. É plausível considerar que a infecção viral tem forte impacto no aporte de energia e no metabolismo em muitos tecidos, contribuindo para o desenvolvimento de sintomas, independentemente da presença de alterações anatômicas/estruturais.^20 , 21^ Em um estudo coorte com 203 pacientes, Willems et al.^22^ encontraram envolvimento de células endoteliais, atividade de coagulação e inflamação constates, na ausência de disfunção macrovascular três meses após COVID-19. Apesar da plausabilidade biológica, a ocorrência de trombose venosa profunda foi rara em nosso estudo, o que limitou inferências gerais sobre sua importância na predição dos sintomas.

Nossos resultados mostraram que as mulheres apresentaram risco três vezes maior de desenvolverem sintomas cardiopulmonares após a alta hospitalar em comparação aos homens. De fato, dados epidemiológicos relatam uma diferença entre sexos quanto à gravidade da COVID-19, com uma evolução mais favorável da doença nas mulheres em comparação aos homens, independentemente da idade,^23 , 24^ e uma taxa semelhante de infecção por SARS-CoV2 em ambos os sexos.^24^ No entanto, as razões das diferenças entre sexos na COVID-19 não foram elucidadas. As mulheres parecem estar relativamente protegidas da COVID-19 devido a uma resposta imune mais eficaz e uma inflamação sistêmica mais amena, com consequente manifestação moderada da doença e menor pré-disposição ao tromboembolismo.^24^ Esses fatos parecem criar um paradoxo para a frequência mais alta de sintomas cardiopulmonares observada entre as mulheres após a alta hospitalar. Assim, é presumível que outros fatores possam contribuir para esses resultados. Por exemplo, sabe-se que as mulheres são mais propensas a perceberem e relatarem sintomas que os homens.^25^ São necessários outros estudos analisando disparidades entre sexos e potenciais implicações em longo prazo na PACS.

Outro resultado de nosso estudo foi a associação independente entre depressão (avaliada por PHQ-2) e sintomas cardiopulmonares três meses após a alta hospitalar. A maioria da literatura disponível abordando depressão no cenário da COVID-19 utilizou avaliações não estruturadas.^26^ Uma revisão sistemática relatou uma frequência entre 3% e 12% de depressão clínica e/ou sintomas depressivos graves na PACS. Em nosso estudo, 6,9% dos pacientes apresentaram depressão 90 dias após a alta hospitalar, e essa condição foi seis vezes maior nos pacientes com sintomas cardiopulmonares que naqueles sem os sintomas. Como explicar essa associação? Os pacientes com depressão geralmente apresentam queixas e uma percepção errônea sobre seu estado de saúde.^27^ Mazza et al.^28^ relataram que a inflamação sistêmica no basal e sua mudança ao longo do tempo foi preditora de sintomas depressivos três meses após a alta hospitalar. Esses resultados destacam as causas multifatoriais e a relevância potencial da depressão no período pós-COVID-19.

Nosso estudo tem pontos fortes e limitações. No estudo, foram avaliados pacientes consecutivos com avaliação a cegas dos sintomas (sem acesso a dados clínicos durante a internação hospitalar). Esse estudo conduziu uma análise abrangente do impacto da evolução da COVID-19 sobre a PACS, incluindo características demográficas, condições pré-existente, distúrbios mentais, entre outras variáveis importantes. Por outro lado, este foi um estudo unicêntrico, com algumas limitações que merecem ser mencionadas. Alguns pacientes não realizaram TC do tórax ou ecocardiograma transtorácico. Desafios importantes relacionados à disponibilidade desses exames após a alta hospitalar foram enfrentados pelos pesquisadores. Muitos desses pacientes vieram de outros estados e outras cidades, de modo que a avaliação por imagem de curto prazo não foi viável para uma proporção significativa de pacientes. A avaliação da função pulmonar e o exame de ressonância magnética não foram realizados pelas mesmas razões e por custos associados a esses procedimentos. Devido a essa disponibilidade limitada, variáveis de imagens não foram incluídas no modelo multivariado. No entanto, é importante reforçar que vários parâmetros da TC do tórax e de ecocardiograma transtorácico não foram diferentes entre os pacientes com e sem sintomas cardiopulmonares (Tabela S2, material suplementar). Assim, é provável que as variáveis de imagem não teriam relação independente com a presença de sintomas cardiopulmonares aos 90 dias. Outra limitação foi o fato de que muitas variáveis foram coletadas apenas em um momento. Várias avaliações ao longo do tempo seriam interessantes para explorar as mudanças dinâmicas dos sintomas pós-COVID-19. Uma revisão sistemática encontrou que uma grande proporção de pacientes apresentou PACS três a 12 meses após a recuperação da fase aguda da COVID-19.^29^ No entanto, estudos disponíveis sobre a PACS são muito heterogêneos, impedindo avaliações apropriadas dos sintomas ao longo do tempo.^29^ Ainda, não ficou claro se algumas condições clínicas, como a depressão, eram uma condição nova ou pré-existente (ou uma exacerbação dessa). A avaliação simultânea de sintomas e da depressão usando procedimentos padrões pode contribuir para explicar o estado clínico atual desses pacientes. A mesma limitação foi observada em estudos anteriores, reforçando a necessidade de uma caracterização adequada dos distúrbios mentais e seu impacto no período pós-COVID-19. Além disso, alguns sintomas cardíacos, como palpitações, não foram analisados nesta investigação, impossibilitando a avaliação de arritmias potenciais. Cansaço/fadiga foi o sintoma mais comum, o qual se trata de um sintoma multifatorial sem ter necessariamente uma relação cardiopulmonar. Contudo, na prática clínica, vários pacientes utilizam fadiga/cansaço como sinônimo de falta de ar/dispneia neste espectro de doenças cardíacas e respiratórias. De fato, Alpert et al. descreveram o sintoma “ *burden* ” (“carga/peso”) e o prognóstico relacionado na insuficiência cardíaca.^30^ Discussão semelhante sobre se o cansaço/fadiga seria um sintoma incapacitante primário nas doenças respiratórias destacou a importância desse sintoma nas interações cardiopulmonares.^31^ Finalmente, nossa entrevista padronizada não classificou os sintomas quanto à gravidade (leve, moderada, grave). Como esperado, todos os pacientes sobreviventes encontravam-se em um momento muito delicado de suas vidas e, nesse contexto, poderiam se sentir cansados ou incomodados por longas entrevistas. Ainda, o sistema de classificação dos sintomas pode não ter sido validado para todos os sintomas avaliados.

## Conclusão

Em conclusão, sintomas cardiopulmonares relacionados à PACS são comuns e multifatoriais. Além dos danos trombóticos, inflamatórios e miocárdicos relacionados à gravidade da COVID-19, ser do sexo feminino e apresentar depressão poderiam explicar a ocorrência de sintomas cardiopulmonares após a alta hospitalar. Esses achados destacam que os sintomas cardiopulmonares relacionados à PACS requerem uma abordagem multifacetada dos pacientes susceptíveis.

## *Material suplementar

Para informação adicional, por favor, clique aqui


