# Supplementary material for: Sintomas Cardiopulmonares Pós-COVID-19: Preditores e Características de Imagem de Pacientes após a Alta Hospitalar
Source: Arq Bras Cardiol. 2023 May 18;120(5):e20220642. [Article in Portuguese] doi: 10.36660/abc.20220642 (PMC10263399; doi:10.36660/abc.20220642)
Supplement: Supplementary file 1 [file 2022-0642-supplemnt.pdf]

ONLINE SUPPLEMENT

Post-COVID-19 CardioPulmonary Symptoms, Predictors and Related Imaging in Patients Discharged from Hospital

Roberto Kalil Filho[1,2]; Roberta Saretta[1]; André Franci[1]; Luciano M. Baracioli[1,2]; Filomena R. B. G. Galas[1]; Juliana S. Gil[1]; Amanda Ferino[1]; Camilla Giacovone[1]; Isabella Oliveira[1]; Johnatan Souza[1]; Vanessa Batista[1]; Augusto Scalabrini-Neto[1]; Livia do Valle Costa[1]; Amanda Danieleto Ruiz[1]; Carla B. Ledo[1]; Teresa Cristina D. C. Nascimento[1]; Luciano F. Drager[1,2]

1. Hospital Sírio Libanês, São Paulo, Brazil
2. Instituto do Coração (InCor), Hospital das Clínicas da Faculdade de Medicina da Universidade de São Paulo, São Paulo, Brazil

Table S1: Characteristics of Patients who had or not available data after hospitalization for COVID-19.

Características	No available data (n=351)	Patients included in the analysis (n=480)	p
Demographic, anthropometric and comorbidities			
Age (years)	57 ± 14	59 ± 14	**0.045**
Male, n (%)	219 (62.4)	324 (67.5)	0.127
Self-reported white, n (%)*	288 (97)	397 (98)	0.789
Body-mass index (kg/m^2)	28.1 (24.8 – 31.2)	27.9 (25.5 – 30.8)	0.881
Alcoholism, n (%)	5 (1.4)	16 (3.3)	0.083
Current smoking, n (%)	15 (4.3)	26 (5.4)	0.452
Hypertension, n (%)	131 (37.3)	202 (42.1)	0.167
Diabetes, n (%)	65 (18.5)	108 (22.5)	0.163
Dyslipidemia, n (%)	93 (26.5)	130 (27.1)	0.850
Previous cardiovascular disease, n (%)	76 (21.7)	142 (29.6)	**0.010**
Previous cerebrovascular disease, n (%)	11 (3.1)	11 (2.3)	0.455
COPD / asthma, n (%)	29 (8.3)	41 (8.5)	0.886
Chronic kidney disease, n (%)	8 (2.3)	13 (2.7)	0.697
Previous diagnosis of anxiety, n (%)	9 (2.6)	13 (2.7)	0.898
Previous diagnosis of depression, n (%)	20 (5.7)	13 (2.7)	**0.029**
Data during hospitalization			
Hospital stay, days (median)	9 (6 - 13)	10 (7 - 16)	**0.016**
Intensive Care Unit, n (%)	74 (21.1)	107 (22.3)	0.194
Mechanical ventilation, n (%)	44 (12.5)	62 (12.9)	0.871
Extracorporeal membrane oxygenation (ECMO), n (%)	5 (1.4)	7 (1.5)	0.968
Pulmonary Embolism, n (%)	9 (2.6)	7 (1.5)	0.252
Deep vein thrombosis, n (%)	6 (1.7)	6 (1.3)	0.583
Dialysis, n (%)	9 (2.6)	4 (0.8)	**0.047**
Major bleeding, n (%)	8 (2.3)	11 (2.3)	0.991
Critical illness polyneuropathy, n (%)	19 (5.4)	19 (4.0)	0.321
Highest level of troponin I, median	0.15 (0.15 – 0.15)	0.15 (0.15 – 0.15)	0.734
Highest level of D dimer, median	605 (368 - 1108)	716 (410 - 1290)	**0.034**
Highest level of C-reactive protein, median (mg/L)	3.29 (0.82 – 8.56)	4.63 (1.52 – 11.54)	**<0.001**
Highest creatinine level (mg/dL)	1.0 (0.85 – 1.15)	1.03 (0.86 – 1.20)	0.140
Antibiotics, n (%)	480 (100)	480 (100)	1.0
Convalescent plasma, n (%)	7 (2.0)	14 (2.9)	0.403
Anticoagulants, n (%)	333 (94.9)	455 (94.8)	0.959
Corticoids, n (%)	254 (72.4)	362 (75.4)	0.321
Chloroquine / hydroxychloroquine, n (%)	64 (18.2)	99 (20.6)	0.391

Table S2: Comparisons of available imaging after hospital discharge in patients with and without cardiorespiratory symptoms. LV: Left Ventricle

Exam	Without cardiorespiratory symptoms	With cardiorespiratory symptoms	p
***Chest CT* (n=122)**			
No lung involvement, n (%)	78 (63.9)	16 (64)	>0.999
Lung involvement 1-25%	39 (32)	8 (32)	
Lung involvement 26-50%	4 (3.3%)	1 (4)	
Lung involvement 51-75%	1 (0.8%)	0 (0)	
Lung involvement >75%	0 (0)	0 (0)	
Pleural effusion, n (%)	2 (1.6)	0 (0)	>0.999
Pericardial effusion, n (%)	1 (0.8)	0 (0)	>0.999
***Transthoracic echocardiogram* (n=98)**			
Left atrum diameter post (mm)	39 (35-43)	36 (32.5-40)	0.033
LV Diastolic diameter post (mm)	48 (45-51)	46 (44-49)	0.105
LV systolic diameter post (mm)	31 (29-33)	29 (28-31.5)	0.119
LV ejection fraction post, %	65 (61-68)	66 (62-68)	0.559
LV ejection fraction reduction ≥5%, n (%)*	19 (19.8)	4 (16.7)	>0.999
LV ejection fraction reduction ≥10%, n (%)*	6 (6.3)	1 (4.2)	>0.999
Alterations in regional LV contractility post, n (%)	10 (10.2)	3 (12.5)	0.718
Pulmonary artery pressure post (mmHg)	28 (24-33)	23 (22-31)	0.177
Pulmonary hypertension (≥35) post, n (%)	7 (7.4)	2 (9.1)	0.676

*** Compared to the in hospital echocardiogram**

Table S3: Multivariate analysis showing variables not independently associated with the cardiorespiratory symptoms (tiredness/dyspnea/respiratory discomfort/cough) 90-days after hospitalization for COVID-19.

	Coefficient	OR	95% Confidence interval		p
**Highest level of D-dimer (ng/mL)**	0.000	1.0000	0.9998	1.0001	0.996
**Hospital stay**	-0.002	0.998	0.957	1.042	0.943
**Quality of life (EuroQol 5)**	0.411	1.508	0.151	15.067	0.726
**Intensive Care Unit**	-0.653	0.521	0.190	1.431	0.206
**Pulmonary sepsis**	-2.296	0.101	0.006	1.830	0.121
**Mechanical ventilation**	-0.309	0.734	0.141	3.819	0.713
**Critical illness polyneuropathy**	-0.837	0.433	0.045	4.194	0.470
**Major bleeding during hospital**	-0.671	0.511	0.027	9.762	0.656
**Simptoms of post-traumatic stress**	0.768	2.156	0.287	16.178	0.455
**Post-traumatic stress disorder**	1.098	2.997	0.871	10.312	0.082
**Anxiety (GAD-2)**	-0.362	0.696	0.186	2.613	0.592

Table S4: Characteristics of patients with and without cardiopulmonary symptoms <u>excluding fatigue/tiredness</u> 90-days after hospitalization for COVID-19.

Characteristics	Without cardiopulmonary symptoms (n=459)	With cardiopulmonary symptoms (n=21)	p
Demographic, anthropometric and comorbidities			
Age (years)	58.8 ± 14.2	61.4 ± 12.9	0.960
Male, n (%)	310 (67.5)	14 (66.7)	0.934
Self-reported white, n (%)*	376 (97.9)	21 (100)	>0.999
Body-mass index (kg/m^2)	27.9 (25.6 – 30.8)	27.5 (24.9 – 30.1)	0.618
Alcoholism, n (%)	14 (3.1)	2 (9.5)	0.151
Current smoking, n (%)	24 (5.2)	2 (9.5)	0.317
Hypertension, n (%)	196 (42.7)	6 (28.6)	0.200
Diabetes, n (%)	103 (22.4)	5 (23.8)	0.795
Dyslipidemia, n (%)	123 (26.8)	7 (33.3)	0.510
Previous cardiovascular disease, n (%)	133 (29.0)	9 (42.9)	0.173
Previous cerebrovascular disease, n (%)	11 (2.4)	0 (0)	>0.999
COPD / asthma, n (%)	39 (8.5)	2 (9.5)	0.698
Chronic kidney disease, n (%)	13 (2.8)	0 (0)	>0.999
Previous diagnosis of anxiety, n (%)	11 (2.4)	2 (9.5)	0.106
Previous diagnosis of depression, n (%)	13 (2.8)	0 (0)	>0.999
Data during hospitalization			
Hospital stay, days (median)	10 (7 – 16)	14 (9 – 32)	**0.011**
Intensive Care Unit, n (%)	99 (21.6)	8 (38.1)	0.103
Mechanical ventilation, n (%)	56 (12.2)	6 (28.6)	**0.041**
Extracorporeal membrane oxygenation (ECMO), n (%)	6 (1.3)	1 (4.8)	0.270
Pulmonary Embolism, n (%)	7 (1.5)	0 (0)	>0.999
Deep vein thrombosis, n (%)	6 (1.3)	0 (0)	>0.999
Dialysis, n (%)	4 (0.9)	0 (0)	>0.999
Major bleeding, n (%)	10 (2.2)	1 (4.8)	0.392
Critical illness polyneuropathy, n (%)	17 (3.7)	2 (9.5)	0.199
Highest level of troponin I (ng/mL)	0.15 (0.15 – 0.15)	0.15 (0.15 – 0.16)	0.796
Highest level of D dimer (ng/mL)	715 (402 - 1290)	743 (455 - 1237)	0.491
Highest level of C-reactive protein (mg/L)	4.57 (1.53 – 11.43)	8.60 (1.44 – 19.84)	0.168
Highest creatinine level (mg/dL)	1.03 (0.86 – 1.20)	0.99 (0.82 – 1.11)	0.462
Antibiotics, n (%)	459 (100)	21 (100)	1.00
Convalescent plasma, n (%)	13 (2.8)	1 (4.8)	0.470
Anticoagulants, n (%)	436 (95.0)	19 (90.5)	0.300
Corticoids, n (%)	344 (74.9)	18 (85.7)	0.262
Chloroquine / hydroxychloroquine, n (%)	93 (20.3)	6 (28.6)	0.519

*** n=405**

Table S5: 90-days data on quality of life, anxiety, depression, post-traumatic stress, post-discharge cardiovascular rehabilitation and readmission by COVID-19 according to the presence or absence of cardiopulmonary symptoms excluding fatigue/tiredness.

**Characteristics**	**Without cardiopulmonary symptoms (n=459)**	**With cardiopulmonary symptoms (n=21)**	**p**
Quality of life (EuroQol 5) *	1 (0.85 – 1.00)	0.85 (0.73 – 1.00	**0.006**
Anxiety (GAD-2), n (%) *	26 (6.8)	3 (18.8)	0.101
Depression (PHQ-2), n (%) *	27 (7.0)	6 (37.5)	**0.001**
Post-traumatic stress disorder, n (%)*	7 (1.8)	2 (12.5)	0.077
Readmission by COVID-19, n (%)	3 (0.7)	0 (0)	>0.999

*** Some patients did not report one or more of these variables.**

Table S6: Multivariate analysis evaluating the independent variables associated with cardiopulmonary symptoms <u>excluding fatigue/tiredness</u> 90-days after hospitalization for COVID-19.

	Coefficient	OR	95% Confidence interval		p
**Age**	-0.022	0.979	0.931	1.029	0.394
**Body mass index (Kg/m^2)**	0.014	1.014	0.903	1.138	0.814
**Smoking (yes)**	0.854	2.349	0.407	13.558	0.340
**COPD / asthma (yes)**	0.057	1.059	0.171	6.554	0.951
**Previous cardiovascular disease (yes)**	0.660	1.935	0.503	7.444	0.337
**Depression (PHQ-2) (yes)**	**2.662**	**14.322**	**4.081**	**50.264**	**<0.001**
**Alcoholism (yes)**	**2.356**	**10.552**	**1.723**	**64.622**	**0.011**
**Days of Hospitalization (per day)**	**0.025**	**1.025**	**1.002**	**1.049**	**0.032**
**Tocilizumab use during hospitalization (yes)**	**4.185**	**65.669**	**3.178**	**1357.003**	**0.007**
**Constant**	-4.049	0.017			0.121
