# Supplementary material for: Sintomas Cardiopulmonares Pós-COVID-19: Preditores e Características de Imagem de Pacientes após a Alta Hospitalar
Source: Arq Bras Cardiol. 2023 May 18;120(5):e20220642. [Article in Portuguese] doi: 10.36660/abc.20220642 (PMC10263399; doi:10.36660/abc.20220642)
Supplement: Translation

# Translation: Post-COVID-19 Cardiopulmonary Symptoms: Predictors and Imaging Features in Patients after Hospital Discharge

**Authors:** Roberto Kalil-Filho, Roberta Saretta, André Franci, Luciano M. Baracioli, Filomena R. B. G. Galas, Juliana S. Gil, Amanda Ferino, Camilla Giacovone, Isabella Oliveira, Johnatan Souza, Vanessa Batista, Augusto Scalabrini, Livia do Valle Costa, Amanda Danieleto Ruiz, Carla B. Ledo, Teresa Cristina D. C. Nascimento, Luciano F. Drager

**Affiliations:** 1 Brazil Hospital Sírio Libanês, São Paulo, SP – Brazil; 2 Brazil Instituto do Coração (InCor), Hospital das Clínicas da Faculdade de Medicina da Universidade de São Paulo, São Paulo, SP – Brazil

**Keywords:** COVID-19, Signs and Symptoms, Sex, Depression

## Abstract

**Background:**

Most of the evidence about the impact of the post-acute COVID-19 Syndrome (PACS) reports individual symptoms without correlations with related imaging.

**Objectives:**

To evaluate cardiopulmonary symptoms, their predictors and related images in COVID-19 patients discharged from hospital.

**Methods:**

Consecutive patients who survived COVID-19 were contacted 90 days after discharge. The Clinic Outcome Team structured a questionnaire evaluating symptoms and clinical status (blinded for hospitalization data). A multivariate analysis was performed to address the course of COVID-19, comorbidities, anxiety, depression, and post-traumatic stress during hospitalization, and cardiac rehabilitation after discharge. The significance level was set at 5%.

**Results:**

A total of 480 discharged patients with COVID-19 (age: 59±14 years, 67.5% males) were included; 22.3% required mechanical ventilation. The prevalence of patients with PACS-related cardiopulmonary symptoms (dyspnea, tiredness/fatigue, cough, and chest discomfort) was 16.3%. Several parameters of chest computed tomography and echocardiogram were similar in patients with and without cardiopulmonary symptoms. The multivariate analysis showed that PACS-related cardiopulmonary-symptoms were independently related to female sex (OR 3.023; 95% CI 1.319-6.929), in-hospital deep venous thrombosis (OR 13.689; 95% CI 1.069-175.304), elevated troponin I (OR 1.355; 95% CI 1.048-1.751) and C-reactive protein during hospitalization (OR 1.060; 95% CI 1.023-1.097) and depression (OR 6.110; 95% CI 2.254-16.558).

**Conclusion:**

PACS-related cardiopulmonary symptoms 90 days post-discharge are common and multifactorial. Beyond thrombotic and markers of inflammation/myocardial injury during hospitalization, female sex and depression were independently associated with cardiopulmonary-related PACS. These results highlighted the need for a multifaceted approach targeting susceptible patients.

**Central Illustration:** : Post-COVID-19 Cardiopulmonary Symptoms: Predictors and Imaging Features in Patients after Hospital Discharge

## Introduction

Coronavirus disease 2019 (COVID-19), a systemic disease caused by the severe acute respiratory syndrome coronavirus 2 (SARS-CoV-2), became one of the biggest challenges to the global population.^1^ On June 30, 2022, 548,101,683 cases had been reported, with 6,337,024 confirmed deaths, which represents an overall mortality rate of 1.16%.^2^ Since the majority of patients survived COVID-19, there has been a growing interest in the potential subacute and long-term effects of COVID-19.^3^ Most reports have focused on the persistent symptoms in patients after acute COVID-19 (collectively described as post-acute COVID-19 syndrome – PACS – or long-COVID),^3 - 5^ while others have reported the impact of COVID-19 on thoracic and cardiac imaging without reference to symptoms.^6 - 8^

Despite this previous evidence, several potential gaps in this timely research area deserve special attention.^9^ First, it is crucial to explore the frequency and the determinants of PACS using a comprehensive approach addressing not only the course of COVID-19 but also other factors such as age, and preexisting conditions including cardiac, pulmonary, and mental disorders after hospital discharge. Moreover, most previous studies on the impact of PACS have not focused on the cardiopulmonary symptoms and not correlated with imaging findings.

The main objective of the study was to assess the magnitude and the independent predictors of cardiopulmonary symptoms related to PACS 90 days after hospital discharge in consecutive patients. In addition, available cardiopulmonary imaging during and after hospitalization were correlated with cardiopulmonary symptoms. The main hypothesis tested was based on the premise that the cardiopulmonary symptoms may have multiple causes, not limited to the severity of COVID-19 during the hospitalization, but also having potential correlation with sex and mental disorders.

## Methods

The data that support the findings of this study are available from the corresponding author upon reasonable request.

The study was reviewed by an Institutional Review Board (IRB) and deemed exempt from IRB oversight (Instituto de Ensino e Pesquisa, IEP, Hospital Sírio Libanês). All data were analyzed in a secure, anonymized database physically separated from the main server. Neither patients nor the public were not involved in the design, or conduct, or reporting, or dissemination plans of our research.

Consecutive hospitalized patients were recruited at the Hospital Sírio Libanês, from March 2020 to April 2021. All patients had a confirmed diagnosis of COVID-19 by the presence of related symptoms and a positive result of a SARS-CoV-2 polymerase chain reaction (PCR) assay for both nasal and pharyngeal swab specimens.

All collected data were reviewed by the study team to assure accuracy. The registry utilized a web-based case report form in the RedCap^TM^plataform (Nashville, TN, US). REDCap is a secure and validate web application for building and managing online surveys and databases (https://www.project-redcap.org/).

### Variables and clinical outcomes collected at the hospital

Participants had their data collected until hospital discharge. Demographic characteristics, medical history, clinical presentation, laboratory results (including the highest levels of troponin I, D-dimer levels, C-reactive protein, and serum creatinine), hospital days, need for mechanical ventilation, extracorporeal membrane oxygenation (ECMO), pulmonary septic shock, pulmonary embolism, deep venous thrombosis, major bleeding, need for dialysis, and medications used during hospitalizations (including antibiotics, anticoagulants, convalescent plasma, corticoids and chloroquine/hydroxychloroquine) were collected for analysis. In addition, data on chest computed tomography (CT) and transthoracic echocardiogram were documented in a subset of patients with available exams. For patients with more than one chest CT during hospitalization, the test indicating the most severe lung involvement was used for the analysis. To quantify the extent of pulmonary abnormalities (total lesions, consolidation, reticulation, and fibrotic-like changes), a semiquantitative CT score was assigned on the basis of the area involved in each of the five lung lobes, as follows: 0, no involvement; 1, less than 5% involvement; 2, 5%–25% involvement; 3, 26%–49% involvement; 4, 50%–75% involvement; and 5, greater than 75% involvement.^10^

### Variables collected after hospital discharge

A standardized questionnaire was applied by the Clinical Outcomes team of the hospital 90 days after hospital discharge. All members of this team had no access to the in-hospital variables. Cardiopulmonary symptoms (self-reported) like dyspnea, tiredness/fatigue, cough, and chest discomfort were considered as cardiopulmonary-related PACS endpoints. Because tiredness/fatigue may represent an unspecific cardiopulmonary symptom, a sub-analysis was performed considering only dyspnea, cough, and chest. In addition, descriptive data on other symptoms such as anosmia, dysgeusia, headache, arthralgia, myalgia, and diarrhea were included in the results.

All patients were evaluated regarding post-discharge cardiovascular rehabilitation program (yes/no), functional status, hospitals readmissions (including for COVID 19-related symptoms), cardiopulmonary events, need of oxygen, and dialysis. In addition, the European Quality of Life Five Dimension (EQ-5D) was applied. This free tool (to register: https://euroqol.org/) encompasses five health domains (mobility, personal care, usual activities, pain/discomfort and anxiety/depression), allowing three response levels, and a visual analogue scale, used for self-assessment of patient’s health. The EQ-5D score ranges from 0 to 1, with 1 representing the best quality of life. The visual analogue scale ranges from 0 to 100, with 100 being “the best imaginable health state and 0 the worst imaginable health state”.^11^

### Post-traumatic stress disorder

Post-traumatic stress disorder related to the COVID-19 hospitalization was objectively evaluated by the following questions: Do you present repetitive and disturbing thoughts, memories, or image regarding the experience of illness/recent hospitalization? Do you experience physical symptoms (e.g., fast heart beating, sweating, difficulty in breathing) when you remember the hospitalization/illness experience?

### Anxiety (Generalized Anxiety Disorder 2-item, GAD-2)

The GAD-2 is a simplified and validated scale used for defining a generalized anxiety disorder.^12^ Each patient was invited to answer the following questions: “Over the last two weeks, how often have you been bothered by the following problems? 1) Feeling nervous, anxious or over your limits; 2) Not being able to stop or control worries”. For each question, one of the following answers was selected: Not at all (0 points); several days (1 point); more than half the days (2 points); nearly every day (3 points). With a cut-off of 3 points (or more) the GAD-2 has a sensitivity of 86% and specificity of 83% for diagnosing generalized anxiety disorder.

### Depression (Patient Health Questionnaire-2, PHQ-2)

The PHQ-2 is an instrument used to investigate the frequency of depressed mood and anhedonia over the past two weeks. Each patient was requested to answer the following questions: Over the last two weeks, how often have you been bothered by the following problems? 1) Little interest or pleasure in doing things; 2) Feeling down, depressed or hopeless. For each question, one of the following answers was selected: Not at all (0 points); several days (1 point); more than half the days (2 points); nearly every day (3 points). The score ranges from 0 to 6. If the score is three or greater, major depressive disorder is likely to be present.^13^

### Statistical analysis

Continuous variables were described by means and standard deviations or median and interquartile range, according to data normality (Shapiro-Wilk test); categorical variables were described using absolute and relative frequencies. In the univariate analysis, the comparisons of patients with and without cardiopulmonary symptoms were performed using the chi-square test for categorical variables and unpaired Student’s t-test/Mann-Whitney for continuous variables (according to the parametric and nonparametric distribution, respectively). Multivariate logistic regression analysis was performed to detect independent predictors of cardiopulmonary symptoms 90 days after hospital discharge considering baseline factors, laboratory findings and post-hospital discharge variables. The variables with a p value <0.2 in the univariate analyses (analysis of one variable at a time) entered in the final model.^14^ In addition, we also considered variables with biological relevance for the main outcomes including age, body mass index, smoking, chronic obstructive pulmonary disease/asthma and previous cardiovascular disease. For anxiety and depression, data from the validated questionnaires (not from self-reported diagnosis) were used for the analysis. The significance level was set at 5%. All analyses were performed with the IBM-SPSS Statistics version 24 (Statistical Package for the Social Sciences, SPSS, Inc, Chicago, IL).

## Results

A total of 883 patients were initially screened. After excluding patients who died during hospitalization and patients transferred to other hospitals, 831 patients had hospital discharge ( Figure 1 ). Characteristics of patients included (n=480) and excluded (n=351) are described in Table S1 (supplementary material). Although several parameters were not different between the two groups, patients included in the analysis were older, had higher rate of cardiovascular diseases, lower frequency of self-reported depression, longer hospital stay, required dialysis more frequently, and had highest levels of D-dimer and C-reactive protein during the hospitalization than patients without post-hospitalization data. Table 1 shows the main characteristics of patients included in the analysis; patients were predominantly men, white, overweight, and had several comorbidities such as hypertension, diabetes, and dyslipidemia. Almost a quarter of them were in the intensive care unit (ICU). As expected for this profile of patients, they had high levels of thrombotic and inflammatory markers.

**Figure 1:** – Study flowchart.

**Table 1:** – Characteristics of patients during hospitalization and after discharge for COVID-19

Characteristics	N=480

Demographic characteristics, anthropometric and comorbidities	
Age (years)	59 ± 14
Male, n (%)	324 (67.5)
Self-reported white, n (%)*	397 (98)
Body mass index (kg/m^2^)	27.9 (25.5 – 30.8)
Alcoholism, n (%)	16 (3.3)
Current smoking, n (%)	26 (5.4)
Hypertension, n (%)	202 (42.1)
Diabetes, n (%)	108 (22.5)
Dyslipidemia, n (%)	130 (27.1)
Previous cardiovascular disease, n (%)	142 (29.6)
Previous cerebrovascular disease, n (%)	11 (2.3)
COPD / asthma, n (%)	41 (8.5)
Chronic kidney disease, n (%)	13 (2.7)
Previous diagnosis of anxiety, n (%)	13 (2.7)
Previous diagnosis of depression, n (%)	13 (2.7)

**Data during hospitalization**	
Hospital stay, days	10 (7 - 16)
Intensive Care Unit, n (%)	107 (22.3)
Mechanical ventilation, n (%)	62 (12.9)
Extracorporeal membrane oxygenation (ECMO), n (%)	7 (1.5)
Pulmonary Embolism, n (%)	7 (1.5)
Deep vein thrombosis, n (%)	6 (1.3)
Dialysis, n (%)	4 (0.8)
Major bleeding, n (%)	11 (2.3)
Critical illness polyneuropathy, n (%)	19 (4.0)
Highest level of troponin I (ng/mL)	0.15 (0.15 – 0.15)
Highest level of D dimer (ng/mL)	716 (410 - 1290)
Highest level of C-reactive protein (mg/L)	4.63 (1.52 – 11.54)
Highest creatinine level (mg/dL)	1.03 (0.86 – 1.20)
Antibiotics, n (%)	480 (100)
Convalescent plasma, n (%)	14 (2.9)
Prophylactic or therapeutic anticoagulants, n (%)	455 (94.8)
Corticoids, n (%)	362 (75.4)
Chloroquine / hydroxychloroquine, n (%)	99 (20.6)

* n=405. COPD: chronic obstructive pulmonary disease.

The prevalence of patients with any symptoms was 32.1% (n=154) and of patients with PACS-related cardiopulmonary symptoms at 90 days was 16.3% (n=78). The most common symptom was tiredness, followed by respiratory discomfort, cough, and dyspnea. Detailed distribution of symptoms is reported in Figure 2 . Patients with cardiopulmonary symptoms at 90 days had a higher frequency of depression, longer hospitalization, higher ICU and mechanical ventilation requirements, higher rate of critical illness polyneuropathy and higher C-reactive protein levels during hospitalization compared to patients without these symptoms ( Table 2 ). Table 3 presents data on structured questionnaires, post-discharge cardiovascular rehabilitation and readmission by COVID-19. Patients with cardiopulmonary symptoms presented worse quality of life, higher rate of anxiety (GAD-2), depression (PHQ-2), and post-traumatic stress disorder than patients without cardiopulmonary symptoms. No difference was observed in the rate of hospital readmission due to COVID-19.

**Figure 2:** – Frequency of symptoms reported by the patients 90 days after hospital discharge for COVID-19.

**Table 2:** – Characteristics of patients with and without cardiopulmonary symptoms (tiredness/dyspnea/respiratory discomfort/cough) 90-days after hospitalization for COVID-19

Characteristics	Without cardiopulmonary symptoms (n=402)	With cardiopulmonary symptoms (n=78)	p
Demographic, anthropometric and comorbidities			
Age (years)	58.6 ± 14.7	60.0 ± 11.4	0.311
Male, n (%)	278 (69.2)	46 (59.0)	0.079
Self-reported white, n (%)*	329 (98.2%)	68 (97.1)	0.363
Body mass index (kg/m^2^)	27.8 (25.6 – 30.8)	28.1 (24.3 – 30.4)	0.477
Alcoholism, n (%)	12 (3.0)	4 (5.1)	0.309
Current smoking, n (%)	23 (5.7)	3 (3.8)	0.784
Hypertension, n (%)	172 (42.8)	30 (38.5)	0.479
Diabetes, n (%)	87 (21.6)	21 (26.9)	0.307
Dyslipidemia, n (%)	108 (26.9)	22 (28.2)	0.808
Previous cardiovascular disease, n (%)	118 (29.4)	24 (30.8)	0.802
Previous cerebrovascular disease, n (%)	10 (2.5)	1 (1.3)	>0.999
COPD / asthma, n (%)	32 (8.0)	9 (11.5)	0.301
Chronic kidney disease, n (%)	11 (2.7)	2 (2.6)	>0.999
Previous diagnosis of anxiety, n (%)	9 (2.2)	4 (5.1)	0.241
Previous diagnosis of depression, n (%)	8 (2.0)	5 (6.4)	0.044

**Data during hospitalization**			
Hospital stay, days	10 (7 – 15)	12 (8 – 22)	0.003
Intensive Care Unit, n (%)	83 (20.6)	24 (30.8)	0.049
Mechanical ventilation, n (%)	43 (10.7)	19 (24.4)	0.001
Extracorporeal membrane oxygenation (ECMO), n (%)	5 (1.2)	2 (2.6)	0.318
Pulmonary Embolism, n (%)	5 (1.2)	2 (2.6)	0.318
Deep vein thrombosis, n (%)	3 (0.7)	3 (3.8)	0.086
Dialysis, n (%)	3 (0.7)	1 (1.3)	0.509
Major bleeding, n (%)	7 (1.7)	4 (5.1)	0.086
Critical illness polyneuropathy, n (%)	12 (3.0)	7 (9.0)	0.022
Highest level of troponin I (ng/mL)	0.15 (0.15 – 0.15)	0.15 (0.15 – 0.16)	0.149
Highest level of D dimer (ng/mL)	705 (391 - 1289)	754 (458 - 1300)	0.122
Highest level of C-reactive protein (mg/L)	4.23 (1.39 – 10.16)	8.31 (2.52 – 18.98)	0.006
Highest creatinine level (mg/dL)	1.04 (0.87 – 1.20)	0.99 (0.83 – 1.19)	0.271
Antibiotics, n (%)	402 (100)	78 (100)	1.00
Convalescent plasma, n (%)	10 (2.5)	4 (5.1)	0.259
Anticoagulants, n (%)	381 (94.8)	74 (94.9)	>0.999
Corticoids, n (%)	299 (74.4)	63 (80.8)	0.230
Chloroquine / hydroxychloroquine, n (%)	80 (19.9)	19 (24.4)	0.373

* n=405; COPD: chronic obstructive pulmonary disease.

**Table 3:** – Data on quality of life, anxiety, depression, post-traumatic stress, cardiovascular rehabilitation 90 days after hospital discharge, and readmission for COVID-19 according to the presence or absence of cardiopulmonary symptoms (tiredness/dyspnea/respiratory discomfort/cough)

Characteristics	Without cardiopulmonary symptoms (n=402)	With cardiopulmonary symptoms (n=78)	p
Quality of life (EuroQol 5) *	1 (0.85 – 1.00)	0.85 (0.73 – 1.00	<0.001
Anxiety (GAD-2), n (%) *	18 (5.4)	11 (16.9)	0.003
Depression (PHQ-2), n (%) *	18 (5.3)	15 (23.1)	<0.001
Post-traumatic stress disorder, n (%)*	4 (1.2)	5 (7.7)	0.013
Readmission by COVID-19, n (%)	2 (0.5)	1 (1.3)	0.413

* Some patients did not report these conditions.

In a subset of patients with available CT scans, most patients showed no lung abnormalities after discharge ( Figure 3 ). Interestingly, several parameters of chest CT and transthoracic echocardiogram were not different between patients with and without cardiopulmonary symptoms (Table S2, supplementary material).

**Figure 3:** – Percentage of lung involvement in patients with COVID-19 during hospital say and after discharge.

Multivariate analysis showed that PACS-related cardiopulmonary symptoms were independently associated with female sex, deep venous thrombosis during the hospital, the highest levels of troponin I and C-reactive protein, and depression (PHQ-2) ( Table 4 ). The other variables included in the model were not significant (Table S3, supplementary material).

**Table 4:** – Multivariate analysis evaluating the independent variables associated with cardiopulmonary symptoms (tiredness/dyspnea/respiratory discomfort/cough) 90 days after hospital discharge for COVID-1

	Coefficient	OR	95% Confidence interval		p
Age (per year)	0.001	1.001	0.999	1.003	0.355
Body mass index (for each 1kg/m^2^)	0.005	1.005	0.999	1.010	0.081
Gender (female)	1.106	3.023	1.319	6.929	0.009
Smoking (yes)	0.615	1.850	0.358	9.568	0.463
COPD / asthma (yes)	0.402	1.495	0.465	4.811	0.500
Previous cardiovascular disease (yes)	0.478	1.612	0.743	3.499	0.227
Deep venous thrombosis (yes)	2.617	13.689	1.069	175.304	0.044
Highest level of troponin I (ng/mL)	0.304	1.355	1.048	1.751	0.020
Highest level of C-reactive protein (mg/L)	0.058	1.060	1.023	1.097	0.001
Depression (PHQ-2) (yes)	1.810	6.110	2.254	16.558	<0.001
Constant	-5.444				<0.001

COPD: chronic obstructive pulmonary disease.

A sub-analysis excluding tiredness as a cardiopulmonary symptom revealed that only 4.4% of patients had dyspnea, cough, or chest discomfort at 90 days (Tables S4-5, supplementary material). Multivariate analysis showed that depression, alcoholism, days of hospitalization and tocilizumab use during hospitalization were independently related to one or more of these symptoms (Table S6, supplementary material).

## Discussion

This study evaluating post-acute COVID-19 cardiopulmonary symptoms 90 days after hospital discharge in consecutive patients shows the following results: 1) PACS-related cardiopulmonary symptoms are presented in 16% of patients; however, when tiredness/fatigue was not considered in the analysis, less than 5% reported dyspnea, cough or chest discomfort; 2) no significant changes were observed in left ventricular function, and most patients showed a normal CT scan in the follow-up. Interestingly, there were no differences in several imaging parameters between patients with and without cardiopulmonary symptoms; 3) the cause of PACS-related cardiopulmonary symptoms is multifactorial. Beyond thrombotic, inflammatory, and myocardial injury markers related to the COVID-19 severity during hospitalization, female sex and the presence of depression are possible explanations for the symptoms (Central Figure). Taken together, our study underscores the need to identify susceptible phenotypes associated with PACS-related cardiopulmonary symptoms for defining preventive strategies using a multifaceted approach.

COVID-19 is frequently associated with a myriad of inflammatory and pro-thrombotic stimuli contributing to a poor prognosis during the hospital phase.^15 , 16^ However, their impact on post-COVID-19 recovery is still unclear. In our study, deep venous thrombosis and increased troponin I and C-reactive protein levels during hospitalization were independently associated with cardiopulmonary symptoms. Previous investigations have suggested that the risk of hospital-associated venous thromboembolism in COVID-19 patients seems to extend over 90 days after discharge starting from the time of admission.^17 - 19^ These results suggest potential carry-over effects that may contribute to the cardiopulmonary symptoms in the short-term follow-up. However, the precise reasons for the relationship between these in-hospital markers and cardiopulmonary symptoms are not entirely clear. Our sub-analysis revealed that several imaging findings (including the degree of lung involvement, lung congestion, pleural and pericardial effusion, left ventricular ejection fraction, alterations in regional left ventricular contractility, pulmonary artery pressure, and the presence of pulmonary hypertension) were not different between patients with and without cardiopulmonary symptoms (Table S2, supplementary material). In this scenario, structural changes promoted by deep venous thrombosis (all of them were appropriately treated during hospitalization) might not fully explain the symptoms related. It is conceivable that the viral infection strongly impacts energy supply and dysregulates metabolism in many tissues favoring symptoms regardless of the presence of anatomical/structural changes.^20 , 21^ Supporting this, in a cohort study including 203 patients, Willems et al.^22^ found sustained endothelial cell involvement, coagulation activity, and inflammation without macrovascular dysfunction three months after COVID-19. Despite the biological plausibility, deep vein thrombosis was rare in our study, limiting general inferences about its importance in predicting symptoms.

Our results revealed that women had a 3-fold higher risk of having cardiopulmonary symptoms after hospital discharge than men. Epidemiological data report a sex difference in the severity of COVID-19, with a more favorable course of the disease in women compared to men regardless of age,^23 , 24^ and similar rate of SARS-CoV-2 infection in both sexes.^24^ However, the reasons for sex differences in COVID-19 have not been fully explained. Women appear to be relatively protected from COVID-19 because of a more effective immune response and a less pronounced systemic inflammation, with consequent moderate clinical manifestations of the disease, and a lower predisposition to thromboembolism.^24^ These facts seem to create a paradox for the higher frequency of cardiopulmonary symptoms observed in females after hospital discharge. It is conceivable that other factors may contribute to this finding. For example, it is known that women are more likely to perceive and report symptoms than men.^25^ Further studies analyzing sex disparities and potential long-term implication in the PACS are warranted.

Another finding from our study was the independent association between depression (evaluated by the PHQ-2) and cardiopulmonary symptoms three months after hospital discharge. Most of the available literature addressing depression in the COVID-19 scenario used non-structured evaluations.^26^ A systematic review reported that the frequency of clinically significant depression and/or severe depressive symptoms ranged from 3 to 12% in PACS. In our study, 6.9% presented depression 90 days after hospital discharge, and this condition was 6-fold higher in patients with cardiopulmonary symptoms than those without them. How to explain this association? Patients with depression usually have frequent complaints and misperceptions about their health status.^27^ Interestingly, Mazza et al.^28^reported that systemic inflammation at baseline and their change over time were shown to predict depressive symptoms at three months post-discharge. These findings highlight the multifactorial causes and the potential relevance of depression in the post-COVID period.

The present study has strengths and limitations to be addressed. This investigation evaluated consecutive patients with blinded assessment of symptoms (without access to clinical data during hospital stay). This study performed a comprehensive analysis of the impact of the course of COVID-19 on PACS including demographic characteristics, preexisting conditions, mental disorders, among other relevant variables. On the other hand, this was a single-center study with some limitations worth noting. Some patients did not perform chest CT or transthoracic echocardiogram. Significant challenges related to the availability to perform these evaluations after hospital discharge were faced by the investigators. Many of these patients came from other cities/estates making this short-term imaging evaluation not feasible for a significant proportion of patients. Pulmonary function evaluation and magnetic resonance imaging were not performed due to the same reasons and related costs associated with these additional procedures. Because of this limited availability, imaging variables were not included in the multivariate model. However, it is important to reinforce that several parameters of chest CT and transthoracic echocardiogram were not different in patients with and without cardiopulmonary symptoms (Table S2, supplementary material). Therefore, the imaging variables would probably not be independently related to the presence of cardiopulmonary symptoms at 90 days. Another limitation is that several variables were collected at one time point. Multiple evaluations over time would be interesting to explore the dynamic changes of post-COVID-19 symptoms. A systematic review found that a large proportion of patients experience PACS three to 12 months after recovery from the acute phase of COVID-19.^29^However, available studies on PACS are highly heterogeneous preventing appropriate evaluations of symptoms over time.^29^It is unclear whether some clinical conditions like depression was a preexisting condition, new-onset, or represented an exacerbated condition. Simultaneous evaluation of symptoms and depression using standard procedures may contribute to explain the current clinical status of these patients. The same limitation has been observed in previous investigations, reinforcing the need for appropriate characterization of mental disorders and their impact in the post-COVID-19 period. Besides, some cardiac symptoms like palpitations were not captured in this investigation, which made it impossible to assess potential arrhythmias. Tiredness/fatigue was the most common symptom, which, *per se* is a multifactorial symptom and does not necessarily have a cardiopulmonary connection. However, in clinical practice, several patients use fatigue/tiredness as synonymous of short of breath/dyspnea in the spectrum of cardiac and respiratory diseases. Indeed, Alpert et al. described the symptom burden (including tiredness/fatigue) and the related prognosis in heart failure.^30^Similar discussion regarding tiredness/fatigue as a primary disabling symptom in chronic respiratory diseases highlighted the importance of this symptom in the cardiopulmonary interactions.^31^Finally, our standardized interview did not grade any symptoms according to severity (mild, moderate and severe). As expected, all surviving patients were in a very delicate time of their lives and in this context, participants may become overwhelmed or annoyed by long interviews. In addition, the grading system may not have an appropriate validation for all symptoms.

## Conclusion

In conclusion, PACS-related cardiopulmonary symptoms are common and multifactorial. Beyond thrombotic, inflammatory, and myocardial damage related to the COVID-19 severity, being female and having depression may explain the occurrence of COVID-19-related cardiopulmonary symptoms after discharge. These findings underscore that cardiopulmonary-related PACS require a multifaceted approach targeting susceptible patients.

## *Supplemental Materials

For additional information, please click here.
